# The compartmentalised nature of neuronal mitophagy: molecular insights and implications

**DOI:** 10.1017/erm.2022.31

**Published:** 2022-09-16

**Authors:** Fivos Borbolis, Konstantinos Palikaras

**Affiliations:** 1 Department of Physiology, School of Medicine, National and Kapodistrian University of Athens, Zografou, Greece; 2 Department of Biology, University of Padova, Padua, Italy

**Keywords:** Cell death, energy metabolism, mitochondria, mitophagy, neurodegeneration, neuron

## Abstract

The maintenance of a healthy mitochondrial network and the ability to adjust organelle population in response to internal or external stimuli are essential for the function and the survival of eukaryotic cells. Over the last two decades several studies have demonstrated the paramount importance of mitophagy, a selective form of autophagy that removes damaged and/or superfluous organelles, in organismal physiology. Post-mitotic neuronal cells are particularly vulnerable to mitochondrial damage, and mitophagy impairment has emerged as a causative factor in multiple neurodegenerative pathologies, including Alzheimer's disease and Parkinson's disease among others. Although mitochondrial turnover is a multifaceted process, neurons have to tackle additional complications, arising from their pronounced bioenergetic demands and their unique architecture and cellular polarisation that render the degradation of distal organelles challenging. Mounting evidence indicates that despite the functional conservation of mitophagy pathways, the unique features of neuronal physiology have led to the adaptation of compartmentalised solutions, which serve to ensure seamless mitochondrial removal in every part of the cell. In this review, we summarise the current knowledge concerning the molecular mechanisms that mediate mitophagy compartmentalisation and discuss their implications in various human pathologies.

## Introduction

Mitochondria are highly dynamic eukaryotic organelles, known as the major source of cellular ATP, generated through the tricarboxylic acid cycle and oxidative phosphorylation (OXPHOS). Nonetheless, they are also involved in other essential cellular processes, including calcium (Ca^2+^) homoeostasis, iron metabolism and the maintenance of redox balance among others (Refs 1–3). Therefore, the maintenance of proper mitochondrial function is pivotal for cellular homoeostasis and viability, and cells have evolved a wide arsenal of quality control mechanisms to sense and respond to aberrations in their activity. Such mechanisms include the control of mitochondrial biogenesis, the mitochondrial unfolded protein response, the integrated stress response, fission and fusion events (collectively termed mitochondrial dynamics) and the selective autophagic removal of damaged or superfluous organelles via mitophagy. The latter has gained a lot of focus, as it has been shown to be an important contributor to key life processes, such as cellular homoeostasis, stress response and differentiation, as well as organismal development, survival, healthspan and longevity. Especially in neurons, mitophagy defects have been associated with a variety of pathological conditions, including Alzheimer's Disease (AD), Parkinson's Disease (PD), Huntington's Disease (HD) and Amyotrophic Lateral Sclerosis (ALS) among others (Table 1) (Refs 4, 5).

**Table 1.** Mitophagy pathways or molecules affected in major neurodegenerative diseases

Disease	Mitophagy pathway/molecule	Reference
Parkinson's Disease	↓PINK1 activity	(Ref. 197)
	↓Parkin activity	(Ref. 198)
	↑LRKK2 activity	(Ref. 110)
	↓Miro translocation to Mito-ER contact sites	(Ref. 199)
Alzheimer's Disease	↓PINK1/Parkin proteins in mitochondria	(Refs 200, 201)
	↓NIX, BNIP3, FUNDC1, OPTN mRNA levels	(Ref. 202)
	↓DISC1, BCL2L13, FUNDC1, PINK1 protein levels	(Refs 176, 187)
Huntington's Disease	↓OPTN, SQSTM1, NBR1 interaction with LC3	(Ref. 42)
	↑DRP1 activity	(Ref. 203)
Amyotrophic Lateral Sclerosis	↓TBK1 activity	(Ref. 43)
	↓OPTN and SQSTM1 interaction with Ub or LC3	(Refs 44–46)
	↓SQSTM1 promoter activity	(Ref. 47)
	↑DRP1 protein levels	(Ref. 204)
	↓Miro protein levels	(Ref. 205)

↑ – increased compared to control, ↓ – decreased compared to control.

Neurons are terminally differentiated, highly polarised cells with distinct subcellular compartments; small cell body, long axonal processes, multiple dendritic branches and numerous synapses. While they are overall energy-demanding cells, synapses are considered to be the major sites of energy consumption, and the positioning of mitochondria near them is required for the maintenance of neurotransmission (Refs 6, 7). At the same time, tight regulation of local Ca^2+^ fluxes is essential for neuronal function and the maintenance of axon potential. To deal with different mitochondrial demands between compartments, neurons rely heavily on the binding of mitochondria to cytoskeletal components that can mediate both local movements and bidirectional transport to or from distal processes. Long-distance transport is mainly performed along precisely oriented microtubule tracks, and mediated by motor proteins that bind mitochondria either directly or indirectly via the action of adaptor molecules (Ref. 8). In axonal processes, microtubules are strictly organised with their plus-ends oriented away and their minus-ends towards the soma (Ref. 9). Therefore, binding of mitochondria to kinesin motor proteins drive anterograde transport, while binding to dynein motor proteins promote retrograde movement. Conversely, movement along microtubules is inhibited by the action of protein-tethers that anchor mitochondria on cytoskeletal components and immobilise them (Ref. 8). Among those, syntaphilin (SNPH) mediates the immobilisation of mitochondria to axonal processes, and its release has been suggested to remobilise stressed mitochondria and retrieve them back to the soma (Refs 10, 11). Although in dendrites microtubule polarity is mixed to a degree that depends on neuronal type, species and the position within the dendrite, mitochondrial transport can be performed using the same molecular mechanisms (Ref. 9). On the other hand, short range distribution and docking of mitochondria seems to be primarily mediated by myosin/actin-based mechanisms (Refs 12–14).

The ability to transport healthy and damaged mitochondria among their compartments liberates neuronal cells from the necessity to locally deploy all available quality control mechanisms in order to sustain mitochondrial homoeostasis. Instead, the bulk of mitochondria quality control can be performed in the soma and proximal regions, where transcripts of relevant nuclear genes are readily available, while organelle transport can mediate their gathering and redistribution according to subcellular demands. However, accumulating evidence suggests that similar events can also occur distally. Mitochondrial biogenesis for instance has been assumed to be restricted in the soma, but recent data argue in favour of local mitochondrial replication, away from the cell body (Ref. 15). Similar implications have been stipulated in the case of mitophagy, but so far data have been contradictory. This review aims to summarise the current knowledge concerning the compartmentalisation of mitophagy in neurons and discuss the associated mechanisms, along with their possible implications in human pathologies.

## Mitophagy mechanisms

The termed mitophagy was first introduced in 2005 to describe the selective removal of mitochondria by the autophagic machinery, which includes three basic steps: the recognition of damaged organelles, the generation of a nascent double-layered autophagic membrane around them and the delivery of the resulting autophagosomes to lysosomes for degradation. (Ref. 16). Since then, a lot of effort has been put in the investigation of the molecular pathways that mediate mitochondrial elimination and govern its regulation, with special interest in the signals that classify an organelle as damaged. Accumulating evidence underlines the existence of multiple mitophagy regulators that respond to various intracellular and/or environmental stimuli and often communicate with each other to exert a fine-tuned mitochondrial quality control. Depending on the involvement of ubiquitin molecules, these pathways can be classified in two large categories: ubiquitin-dependent and ubiquitin-independent mechanisms.

## Ubiquitin-dependent mechanisms

**Fig. 1.**
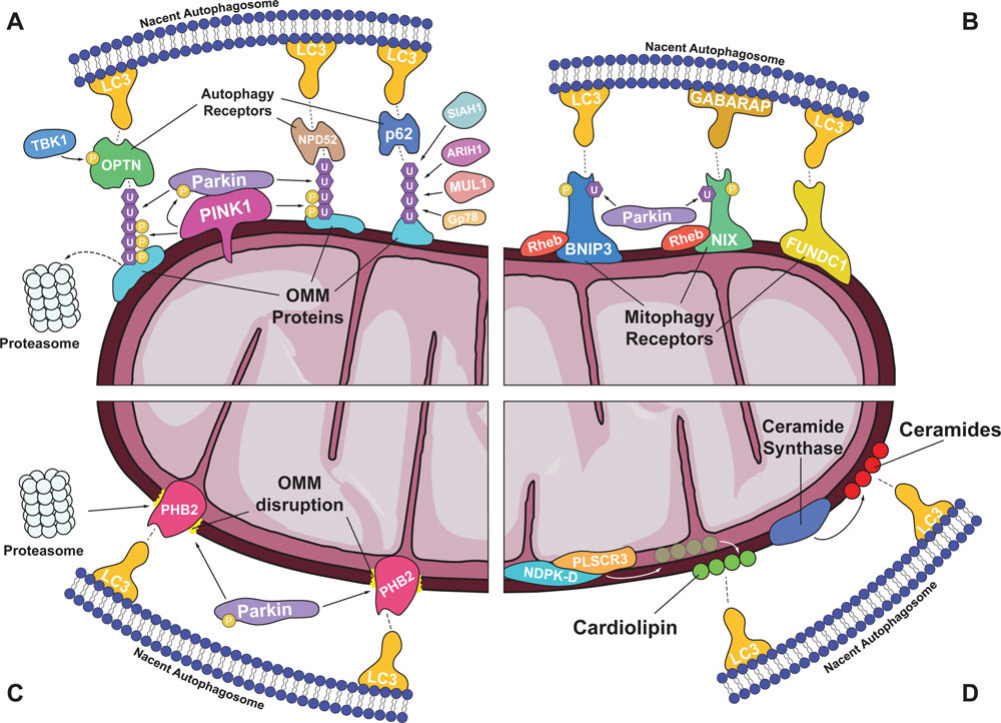
Overview of mitophagy mechanisms. (A) Ubiquitin-mediated mitophagy. Dissipation of mitochondrial membrane potential leads to the accumulation of PINK1 kinase on the surface of damaged organelles, where it phosphorylates and activates the E3 ubiquitin ligase Parkin. Ubiquitin moieties attached to OMM proteins, either by Parkin or by other ubiquitin ligases (e.g., MUL1, SIAH1, ARIH1 and Gp78), are stabilised by PINK1-mediated phosphorylation, and are ultimately recognised by autophagy receptors (e.g., OPTN, NPD52 and p62) that drive autophagosome formation through their interaction with LC3 protein. The action of such receptors is enhanced by their TBK1-mediated phosphorylation. Alternatively, ubiquitinated OMM proteins can be degraded by the proteasome, altering mitochondrial dynamics and motility to favor mitophagy. (B) OMM receptor-mediated mitophagy. OMM proteins can act as mitophagy receptors (e.g., BNIP3, NIX and FUNDC1) and induce autophagosome formation through their direct interaction with LC3/GABARAP. The activation of most mitophagy receptors involves their phosphorylation and is facilitated by the translocation of Rheb small GTPase to mitochondria. Further induction of their activity can be achieved by their Parkin-mediated ubiquitination, establishing a cross-talk mechanism between different pathways. (C) IMM receptor-mediated mitophagy. IMM proteins (e.g., PHB2) can act as mitophagy receptors by interacting with LC3 upon the disruption of OMM, due to Parkin-mediated ubiquitination and proteasomal degradation of OMM proteins. (D) Lipid-mediated mitophagy. The presence of certain lipids (e.g., cardiolipin and ceramides) on the OMM can induce mitophagy by driving autophagosome formation through their interaction with LC3. Cardiolipin is externalised from the IMM by the action of PLSCR3 and NDPK-D, while ceramides and their analogues are freshly generated on the OMM by ceramide synthases.

The pathway that involves the action of the phosphatase and tensin homologue (PTEN)-induced putative kinase protein 1 (PINK1) and the E3 ubiquitin ligase Parkin, constitutes the most extensively studied ubiquitin-dependent mitophagy signalling cascade (Fig. 1a). Both proteins were originally identified as mitochondrial quality control regulators by early studies in *Drosophila melanogaster*, which reported their function in a common pathway, and their mutated forms have been associated with familial cases of PD and other neurodegenerative diseases (Refs 17–19). Under non-stressed conditions, the serine/threonine kinase PINK1 is imported into the inner mitochondrial membrane (IMM) of healthy organelles, where it is proteolytically cleaved, predominantly by PARL protease (Refs 20, 21). Its truncated form is then released to the cytoplasm, where it is rapidly ubiquitinated and degraded by the proteasome, ultimately resulting in low basal protein levels (Refs 20, 21). However, the dissipation of membrane potential in damaged organelles impairs mitochondrial import, leading to the accumulation of PINK1 on the outer mitochondrial membrane (OMM) (Refs 20, 21). In turn, PINK1 phosphorylates cytoplasmic Parkin, stimulating its translocation to the mitochondrial surface and activating its E3 ubiquitin ligase activity (Refs 21–23). At the same time, PINK1 phosphorylates pre-existing or Parkin-attached ubiquitin molecules on various OMM proteins. Binding of Parkin to such phospho-ubiquitin moieties impairs its autoinhibition and further facilitates its direct activation by PINK1 phosphorylation, establishing a feed-forward mechanism for the generation of ubiquitin chains that amplifies the initial signal and ensures mitophagy execution (Refs 21, 24–27). Once activated, Parkin mediates the poly-ubiquitination of various OMM proteins, including the fusion-promoting mitofusins (MFNs) and the kinesin adaptor protein Miro, triggering their proteasomal degradation (Refs 28, 29). The removal of such proteins from the OMM is believed to reduce mitochondrial motility, release mitochondria from contact sites with the endoplasmic reticulum (ER) and promote organelle fission, thereby creating an environment that favours mitophagy (Refs 28, 30, 31). Moreover, PINK1 stimulates the activity of the fission-promoting dynein related protein 1 (DRP1) to induce mitochondrial network fragmentation and the subsequent isolation of defective organelles leading eventually to their engulfment by autophagosomes (Ref. 32). However, the diversity of Parkin substrates suggests that the identity of ubiquitinated OMM proteins is of secondary importance compared to the density of ubiquitin moieties for the induction of mitophagy (Ref. 33).

Beyond the action of Parkin, additional E3 ubiquitin ligases have also been reported to mediate ubiquitination of OMM proteins under mitophagy-inducing conditions, such as Gp78, SIAH1, MUL1 and ARIH1 (Refs 34–37). Regardless of the ubiquitination mechanism, polyubiquitinylated proteins on the OMM have been shown to act as an ‘eat me’ signal recognised by several autophagy adaptors, including optineurin (OPTN), nuclear dot protein 52 (NDP52), sequestosome 1 (SQSTM1/p62), TAX1 binding protein 1 (TAX1BP1) and neighbour of BRCA1 gene 1 (NBR1) (Ref. 38) (Fig. 1a). These adaptor proteins interact directly with ubiquitin chains, through their ubiquitin-binding domain, and with microtubule-associated protein 1A/1B-light chain 3 (LC3), through their LC3-interacting region (LIR), driving autophagosome formation around ubiquitin-tagged mitochondria (Refs 38, 39). Phosphorylation by TANK binding kinase 1 (TBK1) can further modulate the activity of such autophagy receptors, enhancing their affinity for both ubiquitin chains and LC3 (Refs 38, 40, 41). Interestingly, the binding of OPTN to ubiquitin stimulates its phosphorylation by TBK1, suggesting the existence of additional feed-forward mechanisms that enhance the recruitment of the autophagosomal machinery to defective organelles (Ref. 41). Such adaptor proteins seem to have a prominent role in HD, since the polyQ tract in mutant huntingtin (HTT) has been reported to impair their interaction with LC3. As a result, cargo recognition downstream of PINK1/Parkin is impaired, leading to the accumulation of damaged organelles and increased oxidative stress, which contribute to disease pathogenesis (Ref. 42) (Table 1). Likewise, mutations that reduce the function or the expression of TBK1, OPTN or SQSTM1 have been reported to play a causative role in mitophagy disfunctions that contribute to the pathogenesis of ALS (Refs 43–47) (Table 1).

Furthermore, both PINK1 and Parkin have been implicated in some manifestations of an alternative mitochondrial quality control mechanism that involves the generation of Mitochondria Derived Vesicles (MDVs), small vesicular carriers that bud from mildly damaged mitochondria. MDVs are considered to participate in the detoxification system, as they have been shown to transport large assemblies of aggregated or oxidised mitochondrial proteins and lipids to peroxisomes, lysosomes or multivesicular bodies for degradation or export (Refs 48, 49). This mechanism for removing damaged mitochondrial content functions independently of autophagy, does not always necessitate mitochondrial fission and results in the targeted removal of damaged mitochondrial parts, instead of entire organelles. Although the exact mechanism of MDV generation is not clear, targeted recruitment of PINK1/Parkin to specific parts of the organelle is thought to represent the first response, aiming to mitigate mitochondrial dysfunction by ejecting damaged molecules, while sustained insults and depolarisation lead to the outspread of PINK1 and Parkin localisation on the entire mitochondrial surface, activating eventually the process of mitophagy (Refs 48, 49). Despite the undisputable ability of the PINK/Parkin pathway to induce mitochondrial clearance, it is important to highlight that most studies concerning the role of both proteins in mitophagy have been performed in experimental systems that overexpress the corresponding genes. Such artificial conditions could create an unrealistic environment that facilitates mitophagy, resulting in findings that do not relate to physiological conditions. In line with this scepticism, PINK1 or Parkin deficient mice do not exhibit substantial phenotypes related to PD, while both proteins appear dispensable for basal mitophagy *in vivo* (Refs 50–53). Nonetheless, their role seems to be important in stress- and age-induced mitophagy (Refs 54–56). Additional work is necessary to delineate the contribution of the this pathway and of other ubiquitin mediated mechanisms in the regulation of mitophagy under various physiological and pathological conditions.

## Ubiquitin independent mechanisms

Besides ubiquitin-dependent mitophagy, recent studies have identified several regulatory mechanisms that drive autophagosome formation around mitochondria without involving ubiquitination. These mechanisms depend on the presence of mitochondrial receptors that are constitutively localised on the OMM and interact directly with LC3 or GABA receptor associated protein (GABARAP) through their LIR motif, to initiate autophagosomal formation and subsequent mitochondrial elimination (Ref. 57) (Fig. 1b). In contrast to the PINK1/Parkin pathway that appears to orchestrate mitophagy after acute organelle damage, mitophagy receptors seem to be mainly involved in the reorganisation of the mitochondrial network in response to metabolic rewiring, due to environmental stress or during cellular reprograming and differentiation. BCL2 interacting protein 3 (BNIP3) and NIP3-like protein X (NIX/BNIP3L) are two mitophagy receptors that were initially identified as cell death-promoting proteins, but later studies revealed their central role in the selective degradation of mitochondria (Ref. 58). The expression levels of both proteins are induced by hypoxia-inducible factor 1*α* (HIF1*α*) and have been found to mediate hypoxia-induced mitophagy (Ref. 59). Additionally, NIX plays a critical role in the autophagic clearance of mitochondria during erythrocyte maturation or somatic cell reprogramming, in the remodelling of the mitochondrial network during differentiation in cardiac progenitor cells or oligodendrocytes of the optic nerve, and in the cytoprotective induction of mitophagy in neurons during recovery from cerebral ischaemia (Refs 60–64). Despite being mainly associated with basal or programmed mitophagic events, NIX has also been implicated in the clearance of depolarised mitochondria, by promoting GABARAP recruitment and binding on its LIR motif (Ref. 65). The mechanism of NIX activation is not well understood, but it has been proposed to involve phosphorylation of serine residues near its LIR motif, which enhances affinity to LC3, as well as the translocation of Rheb small GTPase to mitochondria that promotes the interaction of NIX with LC3, to induce autophagosomal formation (Refs 66–68). On the other hand, C-terminus phosphorylation inhibits NIX dimerisation and severely reduces its ability to drive mitophagy (Ref. 69). Similar to NIX, BNIP3 interacts with Rheb GTPase, which, in turn, phosphorylates its LIR motif and promotes its homodimerisation and the induction of mitophagy (Refs 70–72). Nonetheless, the function of these mitophagy receptors is not redundant, since NIX overexpression is not sufficient to compensate for BNIP3 depletion and activate excessive mitophagy during ischaemic stroke (Ref. 73).

Other examples of mitophagy receptors include FUN14 domain-containing 1 (FUNDC1), BCL2 like 13 (BCL2L13) and FKBP propyl isomerase 8 (FKBP8) (Ref. 74). Similar to NIX and BNIP3, FUNDC1 is also involved in hypoxia-induced mitophagy, although its activation is not mediated by HIF1-dependent transcriptional induction, but rather on post-transcriptional modifications (Refs 75, 76). Beyond hypoxia, additional physiological or stress induced responses have been reported to involve FUNDC1-mediated mitophagy, such as the remodelling of the mitochondrial network during cardiac progenitor cell differentiation, the clearance of paternal mitochondria in the nematode *Caenorhabditis elegans* and the removal of depolarised organelles following exposure to uncoupling agents (Refs 62, 77, 78). BCL2L13 is the functional homologue of yeast Atg32, an OMM protein that is essential for mitochondrial turnover during respiratory growth. Atg32 interacts with the LC3 homologue Atg8 to initiate autophagosome formation, and its activity is stimulated by phosphorylation (Ref. 79). Likewise, FKBP8 was identified as a mitophagy receptor due to its affinity for Atg8 in yeast two hybrid assays, while later work revealed its implication in hypoxia- or iron depletion-induced mitophagy in mammalian cells (Refs 80, 81). Interestingly, NIX, BNIP3 and FUNDC1 facilitate the translocation of Parkin to the mitochondrial surface, while NIX ubiquitination by Parkin promotes the generation of autophagosomes around damaged organelles, suggesting a crosstalk between different mitophagy pathways (Refs 82–85). Moreover, BNIP3 and FUNDC1 can also interfere with mitochondrial dynamics to favour fragmentation of mitochondria and, thereby, promote mitophagy, by recruiting the fission-promoting DRP1 and triggering the dissociation of the optic atrophy 1 (OPA1) complex that mediates fusion of the IMM (Refs 84, 86, 87). Similarly, BCL2L13 and FKBP8 are able to promote mitochondrial fragmentation even in the absence of DRP1, although the function of the latter depends on OPA1 (Refs 79, 81).

Although mitophagy receptors are located on the OMM, similar functions can be performed by molecules that normally reside on the IMM, upon their exposure to the mitochondrial surface. Prohibitin 2 (PHB2) is an IMM protein that induces mitophagy in a Parkin-dependent manner, as its interaction with LC3 is possible only after OMM disruption, which is achieved by Parkin-mediated ubiquitination and subsequent proteasomal degradation of OMM proteins (Refs 88, 89) (Fig. 1c). PHB2 is essential for the clearance of paternal mitochondria in *C. elegans*, while its interaction with p62 and LC3 is required for bile-acid induced mitophagy in cholestatic liver cells (Refs 88, 90). Moreover, PHB2 has been shown to stabilise PINK1 and promote the recruitment of Parkin and OPTN to mitochondria, by negatively regulating the stability and the activity of PARL protease upon membrane potential dissipation or misfolded protein aggregation (Ref. 91). Likewise, Cardiolipin (CL), a mitochondrion-specific phospholipid, is mainly distributed along the IMM, but is externalised to the OMM of damaged organelles and serves as a mitophagy receptor (Refs 92, 93). In contrast to PHB2 though, externalisation of CL is not the result of OMM disruption, but depends on the action of the mitochondrial phospholipid scramblase-3 (PLSCR3) and the hexameric intermembrane space protein complex of mitochondrial nucleoside diphosphate kinase D (NDPK-D) (Ref. 93) (Fig. 1d). Once on the mitochondrial surface, CL induces autophagosome formation by directly interacting with LC3 (Refs 93, 94). Moreover, NDPK-D activity is inhibited by its association with OPA1, while DRP1 directly binds to CL to promote organelle fission, establishing a direct link between CL translocation and mitochondrial dynamics (Refs 94, 95). Accumulating evidence suggests that additional lipids, such as ceramides and their analogues, can function as mitophagy receptors and promote mitochondrial clearance (Refs 96, 97). Indeed, DRP1 activation results in the p17/PERMIT-mediated translocation of newly translated ceramide synthases from the ER to the OMM, through mitochondrial associated membranes, and the subsequent generation of ceramide that induces LC3 recruitment and autophagosome formation (Ref. 98) (Fig. 1d). While ceramides have been mainly associated with cell death in cancer cells, they have also been implicated in the induction of mitophagy in cardiomyocytes during lipotoxicity (Refs 96, 97). Taken together these observations have sketched a complex network of factors and pathways that regulate and mediate the clearance of mitochondria. Significant effort is still necessary in order to unravel the interplay between the numerous receptors and their interconnections with different mitophagy pathways that coordinate organelle degradation under different physiological or pathological conditions.

## Negative regulators of mitophagy

Although recent years have seen the emergence of impaired mitophagy as a common denominator of various human pathologies, it is becoming increasingly evident that excessive organelle clearance can also have detrimental effects on cellular and organismal homoeostasis (Refs 74, 99, 100). This realisation has spurred novel interest for the discovery of proteins that negatively regulate the process. Targeted inhibition of these proteins can serve as a therapeutic approach that enhances mitophagy, while their induction has therapeutic potential in pathological conditions related to unrestrained organelle turnover. Nevertheless, manipulation of such regulators should be approached with great caution, taking into account possible effects stemming from their involvement in other forms of selective or general autophagy, as well as in additional cellular processes.

Concerning ubiquitin-mediated mechanisms, the action of Parkin and other ubiquitin ligases in promoting mitochondrial clearance is antagonised by deubiquitinating enzymes (DUBs), such as USP30, USP35 and USP15, that mediate ubiquitin chain disassembly and reduce the overall ubiquitin content on the mitochondrial surface (Refs 101–103). Interestingly, depletion of UPS30 has been reported to enhance basal mitophagy in a PINK1-dependent but Parkin-independent manner, an observation that implicates its action in the regulation of basal ubiquitin levels, upstream of any mitophagy-inducing phospho-ubiquitination events (Ref. 104). Therefore, UPS30 (and possibly other DUBs) can determine the threshold for mitophagy initiation by reducing the amount of PINK1 substrates on the OMM. Conversely, UPS30 has been identified as a target of Parkin-mediated ubiquitination that leads to its proteasomal degradation (Refs 101, 103), suggesting that the activation of the PINK1/Parkin pathway suppresses counteracting de-ubiquitination mechanisms, to ensure mitophagy robustness and prevent energy waste by repetitive rounds of ubiquitination and de-ubiquitination. It is therefore possible that the local balance between the activity of ubiquitination and de-ubiquitination machineries ultimately determines which organelles will be deemed damaged and proceed to degradation. Not all deubiquitinating enzymes though have a negative impact on mitophagy. USP8 for instance, has been reported to have an opposing function and induce mitophagy by directly de-ubiquitinating Parkin and, thus, promoting its translocation to impaired mitochondria (Ref. 105).

A similar mitophagy-inhibiting role has been described for PTEN-L, the long isoform of phosphatase and tensin homologue (PTEN), which has been shown to counteract PINK1 and dephosphorylate ubiquitin molecules on the OMM, thus inhibiting the activation of Parkin and its translocation to mitochondria (Ref. 106). Since phosphorylated ubiquitin molecules and polyubiquitin chains on the OMM are resistant to hydrolytic removal (Ref. 107), their dephosphorylation by PTEN-L is also expected to promote the action of DUBs and favour the reduction of the overall ubiquitin content on the mitochondrial surface, ultimately serving as a general blockade of the feedforward phospho-ubiquitination loop that activates mitophagy. Moreover, there are several studies that frame the short isoform of PTEN (or canonical PTEN) as a negative regulator of mitophagy, albeit in a more indirect manner, though the modulation of PI3K-AKT or TLR4-JNK signalling, RAB7A activity and AMPK-CREB transcriptional control (Refs 108, 109). Nonetheless, a lot of work is still necessary in order to fully elucidate the role of PTEN in the regulation mitophagy.

Beyond the regulation of mitochondrial ubiquitin content, other proteins have been found to exert a negative effect on mitophagy through different mechanisms. Among them, Leucine-Rich Repeat Kinase 2 (LRRK2) is of particular interest, since LRRK2 mutations are the most common monogenic cause of PD (Ref. 110). Data from FRET imaging have suggested that LRKK2 kinase activity inhibits mitophagy by interfering with protein-protein interactions that involve PINK1 and DRP1 on mitochondria (Ref. 111). Moreover, LRKK2 has been reported to phosphorylate RAB10, a member of the Rab small GTPase family, which contains key regulators of vesicular trafficking (Ref. 112). In turn, unphosphorylated RAB10 was found to bind OPTN and induce its translocation to depolarised mitochondria, facilitating their clearance, a response that was not recapitulated by a RAB10 phosphomimetic variant (Ref. 113). Interestingly, the two most common LRKK2 mutations in PD patients (G2019S and R1441C) enhance RAB10 phosphorylation and exhibit impaired mitophagy, while genetic knockout or pharmacological inhibition of LRKK2 appears sufficient to rescue mitophagy defects in both primary skin fibroblasts derived from PD patients with relevant mutations, and tissues with clinical relevance of a mouse GS2019S model, without affecting general autophagy (Refs 113–115). Additionally, the same mutation has been reported to impair the removal of Miro1 from damaged mitochondria and delay organelle clearance, indicating a more complex role for LRKK2 in the regulation of mitophagy (Ref. 116).

A more indirect role has been described for poly-ADP-ribose polymerase 1 (PARP1), a DNA repair enzyme that consumes NAD^+^ as a substrate for ADP-ribosylation. PARP1 activation depletes cellular NAD^+^ and attenuates the activity of the NAD^+^-dependent deacetylase SIRT1. This impairment of SIRT1 has been shown to reduce the activity of peroxisome proliferator-activated receptor gamma coactivator 1-alpha (PGC-1*α*/PPARGC1A), and thus decrease the expression of the uncoupling protein UPC2. As a result, mitochondrial membrane potential is increased, leading to PINK1 cleavage and the inhibition of mitophagy (Refs 117, 118). An additional negative regulatory mechanism has been described in yeast, where the PP2A-like protein phosphatase Pgp1 counteracts the phosphorylation of Atg32 mitophagy receptor by casein kinase 2 (CK2) to inhibit mitophagy. Accordingly, deletion of Pgp1 results in the constitutive interaction of Atg32 with Atg11, a cytosolic adaptor protein for selective autophagy, and accelerates mitophagy (Ref. 119). Given the functional and structural conservation of the PP2A family from yeast to mammals, it is possible that a similar mechanism restrains the phosphorylation of BCL2L13 or other mitophagy receptors, to negatively regulate mitophagy in mammalian cells. Supporting this notion, mammalian PP2A has been shown to regulate autophagy by dephosphorylating the autophagy activating kinase ULK1 upon amino acid starvation (Ref. 120). However, evidence for its involvement in selective mitophagy is still lacking.

## Mitophagy compartmentalisation in neuronal cells

Although mitochondrial turnover is taking place in nearly all eukaryotic cells, underscoring its pivotal role for cellular and tissue homoeostasis, the post-mitotic nature of mature neurons provides an extra challenge. Dividing cells can reshuffle their mitochondrial content by inducing organelle biogenesis, coupled with cell division to ‘dilute’ dysfunctional organelles with healthy ones in daughter cells. Besides, most tissues have the ability to face a decline in mitochondrial quality surveillance system by inducing the death of problematic cells, and replenish their cell content by stimulating mitosis of healthy ones. These options however do not exist in neurons, which have to maintain the functionality of their mitochondrial network throughout the lifespan of the organism, which can reach several decades. The aforementioned mitophagy pathways have been mostly studied in non-neuronal cells under stressful conditions, leaving many answered questions concerning their implication in basal mitochondrial turnover of neurons, the answers to which are only just starting to emerge. Moreover, the complex architecture of neuronal cells poses additional challenges in mitochondrial clearance that small and simply organised cells do not have to face, since a number of mitochondria is located at distal neuronal compartments, away from the main cell body. Can such distal organelles be locally degraded or do they have travel back to the soma where mature lysosomes are relatively more abundant? Are all neuronal mitochondria subjected to the same quality control mechanisms regardless of their topology, or do different mitophagy pathways prevail in each compartment? The next part of this review summarises our current knowledge concerning these issues.

## Mitophagy in somatodendritic regions

The cell body, along with proximal dendritic regions, are considered to be the main sites of mitophagy in neuronal cells, as there resides the vast majority of mature lysosomes (Refs 121, 122). Indeed, the inhibition of lysosomal activity has been reported to result in the accumulation of organelles in neuronal somata (Ref. 123). Still, it is not clear which is the prevalent pathway that mediates this process, as relevant results have been contradictive. *In vitro* studies in neuronal cell lines and primary neurons have reported that Parkin selectively translocates to depolarised mitochondria in the soma and proximal dendritic regions to induce autophagosomal organelle clearance after exposure to various damaging agents, suggesting that the function of the pathway is conserved across cell types (Refs 123–126). However, *in vivo* data do not support a major role for PINK1 and Parkin in mitochondrial turnover in neuronal cell bodies under physiological conditions. Studies in both mice and *Drosophila* model systems have shown that although basal mitophagy appears to be a widespread phenomenon in the nervous system, it is minimally affected by PINK1 deletion (Refs 52, 53, 127–129). Deficiency of PINK1 or Parkin though has been shown to impair the age-dependent rise in mitophagy events that is observed in *D. melanogaster* dopaminergic neurons, and lead to the accumulation of abnormal mitochondria (Refs 56, 130). This defect could account for the occurrence of PD-like phenotypes in PINK1 and Parkin *Drosophila* mutants, that are not observed in the corresponding murine models, as it is possible that mice deploy additional quality control mechanisms to sustain mitochondrial quality during ageing, which are not activated or even present in flies (Refs 50, 51, 131–133). The apparent discrepancy between *in vivo* and *in vitro* data has been at least partially attributed to the physiologically irrelevant conditions of *in vitro* experimental systems, which include the overexpression of Parkin and the administration of depolarising agents, often coupled with additional insults, to induce mitophagy (Refs 123–126). Interestingly, even under extreme *in vitro* conditions, artificial overexpression of Parkin seems to be a prerequisite for its recruitment to neuronal mitochondria, while the percentage of treated neurons that necessitates Parkin for the removal or the repair of depolarised organelles is rather dispensable (Refs 123, 126, 134). Moreover, in the apparently rare cases of its induction, the kinetics of Parkin-induced mitophagy in neurons are much slower than what has been reported for non-neuronal cells (Refs 123, 126, 134).

A molecular mechanism that could account for this delayed response has recently been described in mouse cortical neurons treated with low dosage of antimycin A, to induce mild depolarisation. Under these conditions, the mitochondrial E3 ubiquitin ligase 1 (MUL1) was reported to ubiquitinate and destabilise MFN2. In turn, MFN2 destabilisation promotes ER-mitochondrial (ER-Mito) tethering, which impairs the translocation of Parkin to slightly damaged organelles and protects them from degradation (Ref. 135). Upregulation of MFN2, due to MUL1 depletion, was shown to result in the release of mitochondria from ER-Mito contact sites and trigger an upsurge in cytoplasmic Ca^2+^ levels that activates calcineurin and stimulates DRP1-mediated mitochondrial fragmentation and clearance (Ref. 135). MUL1 is therefore proposed to act as an early checkpoint that prevents the degradation of neuronal mitochondria under mild stress. On the other hand, results from *D. melanogaster* dopaminergic neurons, mouse cortical neurons and human induced pluripotent stem cells (iPSCs)-derived dopaminergic neurons suggest that under acute exposure to challenging conditions, ubiquitination of MNF2 by either MUL1 or Parkin, and its subsequent degradation, releases mitochondria from the ER and induces mitophagy (Refs 31, 136). These observations are in line with the dual role of MFN2 in the tethering of mitochondria to ER, as it has been reported to both promote and impair their juxtaposition (Refs 137–140). It is possible that a mild decrease of MFN2 levels promotes ER-Mito interactions and inhibits mitophagy, but its depletion below a critical threshold has the opposite result. Although additional interactions could influence the outcome in a cell-type specific manner, evidence for their existence remains obscure. Interestingly, the ubiquitination levels of the kinesin adaptor protein Miro1 were recently correlated with the rate of Parkin translocation to damaged mitochondria in cultured mouse hippocampal neurons, while conditional Miro1 knockout was shown to impair MFN2 ubiquitination and its subsequent degradation in the same cells *in vivo* (Ref. 141). Whether differences in the ubiquitination status of Miro1 between neuronal and non-neuronal cells are involved in the delayed response of neurons to mitochondrial damage remains to be further investigated.

However, the slow kinetics of mitochondrial elimination in neuronal cells extend beyond the rate of Parkin translocation. Recent work in primary hippocampal neurons during mild stress revealed that Parkin-dependent recruitment of OPTN and autophagophore formation around damaged mitochondria occurs at a rate comparable to non-neuronal cells. However, the acidification of the resulting mitophagosomes and the turnover of engulfed organelles is a considerably slow process compared to other cell types (Ref. 142). While the reason behind this delay in acidification remains elusive, the sequestration of damaged organelles is expected to protect neurons by preventing the dissemination of damaged components and the generation of harmful excessive reactive oxygen species (ROS). Further supporting this notion, defective engulfment of damaged mitochondria, due to an ALS-linked dominant OPTN mutation, results in an overall decrease of mitochondrial homoeostasis (Ref. 142). Conversely, the delayed induction of mitophagy could be of vital importance to the survival of neurons, due to their high dependence on OXPHOS, compared to the more glycolytic metabolism of other cell types. Thus, delaying mitophagy would prevent ATP depletion and allow alternative quality control mechanisms to act and repair damaged organelles. Moreover, low ATP levels caused by mitochondrial depolarisation have been proposed to directly impede the energy consuming process of PINK1/Parkin-mediated mitophagy in neurons, by limiting both *de novo* synthesis of PINK1 and the phosphorylation of its targets (Ref. 134).

**Fig. 2.**
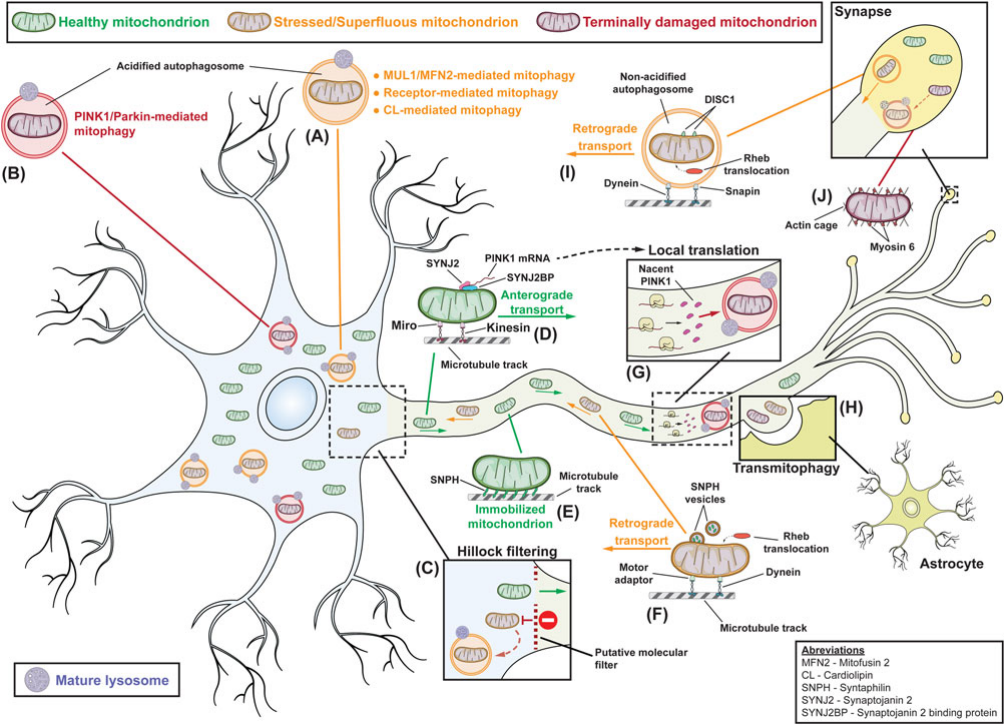
Putative integrated model for the compartmentalisation of mitophagy in neurons. The turnover of stressed or superfluous mitochondria in the main cell body of neurons is predominantly mediated by PINK1-independent mechanisms, which involve the action of mitophagy receptors, cardiolipin and the ubiquitination of MFN2 by MUL1 (A), while terminally damaged organelles are cleared through the PINK1/Parkin pathway (B). Quality control mechanisms at the axonal hillock (C) filter organelles and only allow healthy mitochondria to travel anterograde along microtubule tracks (D), whereas damaged ones are targeted for degradation. Along the axon, the anchoring protein syntaphilin (SNPH) antagonises motor proteins to promote the immobilisation of functional organelles at areas of high mitochondrial demand (E). Rheb small GTPase translocates on stressed axonal mitochondria, which release the anchoring protein SNPH and are remobilised in order to travel retrograde and be degraded in the soma (F). Terminally damaged axonal organelles are degraded locally through the PINK1/Parkin pathway, which is supported by local PINK1 mRNA translation (G). Damaged mitochondria that fail to travel retrograde or be locally degraded can be externalised and cleared by neighbouring astrocytes though the process of transmitophagy (H). At synapses, Rheb translocation induces DISC1-mediated formation of autophagosomes around stressed mitochondria, that remain non-acidified and interact with the dynein adaptor protein Snapin to travel back to the soma (I). Terminally damaged synaptic mitochondria acquire myosin 6, which promotes the assembly of stable actin cages and immobilises organelles to enable their local Parkin-mediated turnover (J). Somatodendritic regions are marked with light blue, axonal regions with light green and synapses with yellow background.

Collectively these observations indicate that neurons reserve PINK1/Parkin-mediated mitophagy as the last resort for the clearance of terminally damaged organelles, while the maintenance of the mitochondrial network under basal conditions or mild stress is mediated by alternative quality surveillance pathways (Fig. 2A, B). These might include the action of the parallel MUL1-MFN2 pathway, as MUL1 overexpression suppresses PINK1 and Parkin mutant phenotypes in *Drosophila* dopaminergic neurons, while its depletion aggravates these phenotypes and leads to synthetic lethality in flies, or to the degeneration of mouse cortical neurons (Ref. 136). Moreover, accumulating evidence also suggests key roles for receptor-mediated mitophagy. CL-mediated removal of damaged organelles has been reported in rat primary cortical neurons after exposure to damaging agents, while CL metabolism in the brain is emerging as a critical factor in the pathogenesis of many neurodegenerative conditions (Refs 93, 143). The function of both NIX and FUNDC1 appears to be involved in mitophagy induction that protects neurons during recovery from cerebral ischaemia (Refs 64, 144), while the upregulation of BNIP3 leads to the toxic overactivation of mitophagy in response to ischaemic stroke, further supporting that proper regulation of different mitophagy mechanisms is vital for neuronal cells viability (Ref. 73). Additionally, NIX has been proposed to mediate the compensatory mechanism that preserves mitochondrial function and clearance in cells of asymptomatic Parkin mutation carriers, while genetic or pharmacological induction of its expression has been shown to restore mitophagy in cells derived from PINK1- or Parkin-related PD patients (Ref. 145). Recently, BNIP3 overexpression was shown to induce mitophagy and prevent the accumulation of dysfunctional organelles in the brain of aged flies (Ref. 146). Intriguingly, neuron-specific upregulation of BNIP3 was able to delay the onset of age-dependent phenotypes in muscle and intestinal cells, suggesting that mitochondrial function in the brain can regulate the rate of organismal ageing through a cell non-autonomous mechanism (Ref. 146). However, the mechanistic insights that mediate such systemic responses are still elusive.

## Mitophagy in axonal processes

The complex architecture and the polarised nature of neurons create an extra challenge in the autophagic turnover of mitochondria; while most enzymatically active and degradative lysosomes are located in their soma, a large portion of their mitochondrial content is located at distal compartments (Refs 121, 122). This problem becomes even more pronounced for motoneurons that can project very long axons, often extending beyond one metre. How do distal organelles meet the lysosomal machinery? Analysis in Purkinje cells of mice expressing the *mito-QC* mitophagy reporter suggests that mitophagic events in axonal projections are extremely scarce, and the wide majority of basal mitochondrial turnover occurs in somatodendritic regions (Ref. 147). Likewise, mild treatment of mouse cortical neurons with depolarising agents has been reported to induce the recruitment of Parkin to mitochondria specifically in cell bodies and proximal regions (Ref. 123), while OPTN translocation to depolarised mitochondria has been shown to transpire mainly in the soma of rat primary hippocampal neurons under mild oxidative stress, with very limited occurrences in distal compartments (Ref. 142). Moreover, *in vivo* studies in mutant PINK1 or Parkin flies did not detect any structural defects that would indicate the accumulation of dysfunctional organelles in the axons of motoneurons when the pathway is impaired, but on the contrary, they reported a reduction in axonal mitochondrial content (Refs 128, 129). At the same time, loss of either protein was shown to disturb the morphology of somatic mitochondria and induce the accumulation of hyperfused organelles in cell bodies of the same neurons (Refs 128, 129). These results support the notion that Parkin-mediated quality control in the soma is essential for the entry of mitochondria in the axons and underline the existence of a ‘gatekeeper’ filtering mechanism at the hillock that only allows organelles with specific qualitative criteria to enter (Fig. 2c). Evidence for the molecular identity of this filter though is yet to be presented.

As far as organelle turnover is concerned, the aforementioned data have shaped a model where damaged mitochondria are transferred back to the soma, where mature lysosomes are predominately located, in order to be degraded. Early evidence in favour of this model comes from a study using the voltage-sensitive dye JC-1 in chicken dorsal root ganglion (DRG) neurons, where axonal mitochondria with low membrane potential were reported to travel towards the cell body, while the inhibition of the electron transport chain (ETC) with antimycin A was shown to increase retrograde mitochondrial transport (Ref. 148). More recent work uncovered a crucial role for the static anchor protein SNPH, which counteracts the motility-promoting function of motor proteins and immobilises mitochondria on specific positions along microtubule tracks (Ref. 149) (Fig. 2d, e). Low-dosage treatment with antimycin A, was shown to induce the release of SNPH from mildly stressed axonal mitochondria of mouse cortical neurons, in the form of cargo vesicles that are generated independently of both Parkin and DRP1 (Ref. 11). In turn, the release of SNPH was shown to remobilise damaged organelles and facilitate their retrograde transport, ultimately mediating the recovery of mitochondrial membrane in axonal organelles (Ref. 11) (Fig. 2f). Moreover, the small GTPase Rheb has been proposed to sense membrane potential dissipation and induce clearance of axonal mitochondria under both physiological and stressed conditions (Ref. 150). Recent studies in mouse cortical neurons indicate that treatment with antimycin A or the depolarising agent CCCP, triggers Rheb translocation to depolarised axonal mitochondria initiating the formation of mitophagosomes and their dynein-mediated transport to the soma, where they are acidified by fusion with degradative lysosomes (Ref. 150). Notably, Parkin is dispensable for Rheb-associated mitophagy initiation within axons, whereas the function of NIX is required, highlighting its pivotal role of in the turnover of distal mitochondria (Ref. 150). In addition to Rheb, the small GTPase Rhes was shown to promote mitochondrial removal in the striatum of mice in response to 3-nitropropionic acid (3-NP), an inhibitor of the respiratory complex II, suggesting that additional small GTPases might perform similar functions in a cell type-specific manner (Ref. 151). A recent study utilised ischaemic mouse primary cortical neurons combined with the fluorescence-shifting mitochondrial protein mitoDentra2 to investigate the fate of axonal mitochondria. Interestingly, retrograde moving axonal organelles were shown to ultimately fuse with lysosomes in the neuronal cell body in response to oxygen and glucose deprivation underscoring that those motile mitochondria are targeted for autophagic clearance upon stress conditions (Ref. 152).

However, there is a series of evidence that puts this model into question. Autophagosomes have been shown to be generated distally, with their maturation taking place within the confinements of axonal processes in mouse DRG neurons (Ref. 153). At same time, delivery of enzymatically active degradative lysosomes from the soma to distal axons has been observed in mouse cortical neurons, rendering the local degradation of axonal organelles a plausible scenario (Ref. 154). Moreover, the assessment of mitochondrial membrane potential in chicken DRG neurons did not uncover any differences between anterograde and retrograde moving axonal organelles (Ref. 155), while treatment with ETC inhibitors and depolarising agents has often been reported to prevent mitochondrial movement, by inducing the degradation of Miro1 (Refs 29, 152, 156). This immobilisation has been proposed to isolate defective mitochondria and promote their in-situ clearance, thus avoiding the dissemination of their damaged components to healthy mitochondrial population via fusion events, or the spreading of excessive ROS to other cellular compartments. Indeed, selective impairment of axonal mitochondria in rat hippocampal neurons either by local administration of antimycin A or by expressing the light-inducible mitochondria-targeted photosensitizer KillerRed, has been shown to trigger local autophagosome formation, engulfing defective axonal mitochondria in a PINK1- and Parkin-dependent manner (Ref. 157). Interestingly, the same study reported that Parkin is not required for the initial remodelling of the mitochondrial network after depolarisation, and its translocation is restricted to a limited subset of damaged organelles. These observations indicate the existence of additional mechanisms that restore mitochondrial health in axons highlighting Parkin availability as a limiting factor for local mitophagy initiation. The latter is in line with the particular needs of the PINK1/Parkin pathway that is expected to necessitate a continuous supply of newly synthesised PINK1 protein from the cell body. However, the rapid cleavage of the protein in healthy mitochondria would make its transport to distal parts of the axon problematic. A mechanism to overcome this challenge was described recently in human iPSCs-derived neurons, where *Pink1* mRNA was found to be tethered on mitochondria via the action of synaptojanin 2 (SYNJ2) and synaptojanin 2 binding protein (SYNJ2BP) (Fig. 2d). Through this tethering, *Pink1* transcripts were shown to hitchhike their way to distal processes and mediate the local translation of nascent PINK1, thereby, enabling the PINK1/Parkin-mediated distal mitophagy (Ref. 158) (Fig. 2g). Interestingly, this mechanism for *Pink1* mRNA distribution appears to be neuron-specific, due to low levels of SYNJ2 expression in non-neuronal cells.

The integration of these data has shaped a proposed model for the degradation of distal mitochondria, according to which basal turnover occurs via their transport to the soma, where they meet with mature lysosomes (Fig. 2f), but severely damaged organelles are immobilised in axons and eventually trigger the PINK1/Parkin machinery that mediates their local engulfment by autophagosomal membranes and their subsequent degradation (Fig. 2g). Further evidence for this model stems from the observations that overexpression of SNPH results in the enrichment of Parkin-positive immobilised mitochondria in distal axonal processes of mouse cortical neurons and inhibits mitochondrial retrograde transport and degradation in the soma after oxygen deprivation (Refs 123, 152). Therefore, it seems that distal mitochondria that carry damage but fail to travel retrograde to the soma will eventually recruit Parkin. However, the scarcity of PINK1/Parkin events predicted by this model fails to explain the absence of organelle accumulation in distal parts of the axon, despite the anterograde bias in the ratio of anterograde to retrograde transport that has been reported in various systems (Refs 29, 159, 160). Moreover, the use of the time-sensitive MitoTimer fluorescent probe in primary mouse hippocampal neurons has revealed the accrual of old proteins in stationary distal mitochondria (Ref. 161). If aged mitochondria do not travel back to the soma, how do they get replenished by new ones? A possible solution could be provided by the cell non-autonomous mechanism of transmitophagy that has been described in mouse retinal ganglion cells (RGCs) and nigrostriatal dopaminergic neurons (DAn) of chemically induced PD rodent models (Refs 162, 163). According to these studies, pools of damaged mitochondria can be transported to adjacent astrocytes, where they get degraded (Fig. 2h). Coupled with the observation that the mitophagy promoting small GTPase Rhes can travel from cell to cell and interact with NIX in the murine striatum, these data suggest the existence of an intercellular mitochondrial quality control mechanism in the nervous system (Ref. 151). Similar conclusions have been recently drawn from co-cultures of murine primary cortical neurons with astrocytes or human iPSCs-derived neurons with astrocyte-like cells, indicating that transmitophagy is a common strategy adopted by various types of neurons (Ref. 164). This alternative way to remove damaged mitochondria from the axons could be essential for neuronal survival under pathological conditions that involve defects in mitophagy. In agreement with this scenario, astrocyte clearance of neuronal mitochondria is induced in co-cultured cells from AD mouse models, or iPSCs from symptomatic AD patients (Ref. 164), probably acting as compensatory mechanism, while the reduced capacity of astrocytes to execute transmitophagy has been suggested to participate in the pathogenesis of PD (Ref. 163). Further investigation on the field of axonal mitochondria clearance is expected to provide valuable information on the pathogenesis and the treatment of neurodegenerative diseases.

## Mitophagy of synaptic organelles

Synaptic dysfunction is a common denominator of many late-onset neurodegenerative disorders and it is believed to have a significant impact on disease progression (Ref. 165). There is a plethora of studies that highlights the contribution of synaptic failure in the manifestations of PD (Ref. 166). At the same time, the toxic effect of Α*β* oligomers on synaptic activity is considered as one of the main events that underlie cognitive deficits related to AD (Ref. 167). The high metabolic status of synapses and their role in neuronal physiology renders the presence of healthy mitochondria essential for their function, due to their role in ATP generation and Ca^2+^ buffering. It is therefore not surprising that dysfunctional mitophagy is starting to emerge as a causative factor of synaptic failure and neuronal cell death (Refs 4, 168). However, the molecular mechanisms that sustain mitochondrial homoeostasis at synapses remain elusive. Evidence from multiple experimental systems suggest that the majority of synaptic mitochondria in mature neurons are stationary and their immobilisation is further enhanced upon synaptic activity (Refs 169–172). This reduced mobility might contribute to the increased wear of synaptic mitochondria, which have been reported to display decreased capacity for energy production and to become more susceptible to Ca^2+^-induced depolarisation with age, compared to axonal organelles (Ref. 173). Such observations suggest that clearance of synaptic organelles requires their remobilisation in order to travel back to the soma, without however excluding the contribution of local degradation mechanisms.

The importance of mitochondria remobilisation for the maintenance of synaptic activity was recently reported in a mouse AD model that exhibits synapse loss at early disease stages due to the expression of mutant human amyloid precursor protein (APP). Observations from synaptic regions in the brain of these animals revealed a marked increase in the number of Rheb-associated mitochondria that display features of early mitophagic events. However, despite the induction of mitophagy, retrograde movement of organelles in the corresponding axons was impaired leading to the accumulation of non-acidified mitophagosomes at synapses (Ref. 150). Interestingly, deficiency of the dynein adaptor protein Snapin was sufficient to mimic this phenotype, while its overexpression was able to alleviate the accumulation of mitophagosomes and mitigate synaptic loss in the brain of AD mice, highlighting the importance of retrograde transport in both physiological and pathological conditions (Ref. 150). A putative role in the clearance of dysfunctional synaptic organelles has also been suggested for disrupted in schizophrenia 1 (DISC1), a protein that is present in multiple subcellular compartments, but localises predominantly at mitochondria, and is enriched in synaptic terminals (Refs 174, 175). DISC1 has been associated with psychiatric disorders and AD pathology, and was recently identified as a potent inducer of mitophagy in response to treatment with Α*β* oligomers or CCCP (Ref. 176). Mechanistically, DISC1 serves as a mitophagy receptor and interacts directly with LC3 through its LIR motif to promote autophagosomal formation (Ref. 176). Moreover, DISC1 overexpression was reported to induce mitophagy and, thereby, prevent both mitochondrial dysfunction and decreased density of dendritic spines upon Α*β* oligomers supplementation in mouse primary cortical neurons (Ref. 176). Notably, DISC1-mediated mitophagy acts as a major contributor of energy homoeostasis at synapses, since its activation alleviates Α*β*-induced impairment of synaptic plasticity and improves cognitive function in a mouse AD model (Ref. 176). Furthermore, DISC1 has been also reported to interact directly with SNPH, inhibit its anchoring ability and, thereby, promote the mobility of axonal organelles (Ref. 177). Therefore, it is tempting to speculate that both Rheb and DISC1 act synergistically to enhance the remobilisation of aged and/or damaged mitochondria that are anchored in synaptic regions, subsequently leading to their degradation through receptor-mediated mitophagy (Fig. 2i). Despite all indications though, direct evidence for the regulation of synaptic mitophagy by DISC1 is still lacking and additional work is required to support such a model.

An opposing role that could promote the local degradation of mitochondria at synaptic regions might be performed by myosin 6, which has been identified as a mitophagy modulator in non-neuronal cells. Particularly, myosin 6 interacts with Parkin-generated poly-ubiquitin chains and promotes the assembly of stable actin cages around damaged mitochondria mediating their isolation from the healthy mitochondrial network and their subsequent removal (Ref. 178). The implication of myosin 6 in mitophagy is further supported by its interaction with established mitophagy receptors that act downstream of Parkin, such as OPTN, NDP52 and TAX1BP1 (Refs 179, 180). Given the well-established role of the protein in synapse formation and function, it is plausible that its recruitment to dysfunctional organelles orchestrates their local Parkin-mediated turnover to sustain synaptic homoeostasis (Ref. 181) (Fig. 2j). Supporting this hypothesis, myosin 6 knockout mice exhibit alterations in synaptic structure and gliosis in the brain, phenotypes related to neurodegenerative disorders (Ref. 182). Although such local turnover events are yet to be confirmed, these observations suggest that despite the contribution of additional regulators, mitophagy at synapses shares the same mechanistic principles with proximal axonal regions, and while retrograde transport is the predominant pathway for the clearance of organelles, severe damage can induce Parkin-mediated local degradation. However, further investigation is deemed necessary to unravel the mechanistic insights of mitophagy at synapses.

## Concluding remarks

A plethora of studies in the past few years has highlighted the essential contribution of mitophagy in the maintenance of a healthy mitochondrial network, a feature of paramount importance for the function and the survival of neuronal cells. Although the basic principles and molecular constituents of mitophagy appear to be mechanistically conserved across cell types, accumulative data has shown that neurons differentiate on the kinetics of the process and have adopted compartment-specific variations, in order to mitigate challenges that arise from their unique structural and functional characteristics. Despite the recent advances in our knowledge though, there are gaps in the implicated molecular interactions and several questions that still remain to be addressed. Is mitophagy organised in the same way in all neurons? Do different subtypes exhibit unique features? Are perturbations in the compartmentalisation of mitophagy related to the pathogenesis of neurodegenerative disorders? Future studies focusing on these subjects will greatly deepen our understanding on neuronal mitophagy and help design targeted therapeutic interventions.

Failure to achieve proper turnover of mitochondria leads to a progressive accumulation of dysfunctional organelles that is considered a hallmark of ageing, as well as a causative factor in many age-related neurodegenerative disorders (Refs 100, 183, 184). Such observations have spurred great interest in the discovery of natural or synthetic compounds that stimulate mitophagy, and indeed pharmacological upregulation of the process has been shown to protect against various neurodegenerative pathologies (Refs 4, 117, 185, 186). Induction of mitophagy by treatment with the microflora-derived metabolite urolithin A (UA) has been reported to protect against Α*β*-induced neurotoxicity and extend the lifespan of the nematode *C. elegans* (Refs 187, 188). Likewise, UA supplementation has been found to inhibit Α*β* and tau pathologies in transgenic AD model mice (APP/PS1), whilst reversing their cognitive defects (Ref. 187). At the same time, the increase of nicotinamide adenine dinucleotide (NAD^+^) levels by direct supplementation or by administration of precursor molecules, such as nicotinamide mononucleotide (NMN) or nicotinamide riboside (NR), has been shown to alleviate pathologies of multiple neurodegenerative disorders, including AD, PD and HD in various model systems (Refs 187, 189, 190). Although data from clinical trials are still insufficient to provide safe conclusions, initial observations suggest that UA, NMN and NR can be safely administered to humans, without the occurrence of adverse side effects, further highlighting their therapeutical potential (Refs 190, 191). Nevertheless, the pathways that are affected downstream of such mitophagy inducing compounds are still largely unexplored. Deciphering their mechanism of action should constitute a major goal in the field, that will maximise their therapeutic potential and improve our perception of mitophagy regulatory mechanisms under both normal and challenged conditions. In this effort, animal models of neurodegenerative diseases and *in vivo* tools for mitophagy assessment, such as mitoRosella, mitoKeima and mito-QC, will be valuable tools.

Despite promising results though, there is significant evidence that a disproportionate reduction of organelle numbers can have detrimental effects in the physiology of neurons and ultimately trigger cell death (Refs 100, 192, 193). Congruently, BNIP3-mediated mitophagy has been reported to promote neuronal cell death both *in vivo* and *in vitro* after ischaemic stroke, while excessive Rhes- and NIX-mediated mitophagy has been shown to cause degeneration of striatum neurons in the brain of 3-NP-treated mice, emulating lesions related to HD (Refs 73, 151, 194). Additionally, exaggerated mitophagy was recently found to mediate mitochondrial depletion from the axons of retinal ganglion cells (RGCs) that express the mutated forms of the IMM fusion promoting protein OPA1 (Ref. 195). In turn, decreased mitochondrial number in the axonal region was identified as the main cause of neurodegeneration that leads to the development of autosomal dominant optical atrophy (ADOA), a genetic disorder characterised by bilateral visual loss in early childhood, and secondary multi-systemic pathologies, which include deafness, ataxia and myopathies (Refs 195, 196). Interestingly, mitochondria were found to co-accumulate with autophagic vesicles close to the axonal hillock of ADOA RGCs. Moreover, inhibition of autophagy or mitophagy was able to restore axonal mitochondrial content in both primary RGCs and *C. elegans* GABAergic neurons modelling ADOA, and protect RGC-specific Opa1 knockout mice from exhibiting visual loss (Ref. 195). These observations indicate that local mitophagy close to the hillock could be part of the putative filtering machinery that ensures the health of axonal organelles, since the degradation of those that fail quality control seems necessary in order to prevent their anterograde travel. Furthermore, the prevention of visual loss by curtailing autophagy suggests that, despite their defects, Opa1 depleted mitochondria are competent to support neuronal function and survival if they are spared from degradation and allowed to travel, rendering the function of a quality-reassuring mechanism detrimental. In this context, a better understanding of the mechanisms that allow or inhibit anterograde mitochondrial transfer along the axon will provide valuable information with therapeutical potential in neurodegenerative conditions. Moreover, such observations make evident that both the dysfunction and the overstimulation of mitophagy can lead to neuronal pathologies and any intervention that aims to interfere with organelle turnover should take into account the importance of maintaining the proper equilibrium between mitochondrial biogenesis and degradation.
